# Dietary Fish Meal Replacement with *Hermetia illucens* and *Tenebrio molitor* Larval Meals Improves the Growth Performance and Nutriphysiological Status of Ide (*Leuciscus idus*) Juveniles

**DOI:** 10.3390/ani12101227

**Published:** 2022-05-10

**Authors:** Natalia Homska, Joanna Kowalska, Joanna Bogucka, Ewa Ziółkowska, Mateusz Rawski, Bartosz Kierończyk, Jan Mazurkiewicz

**Affiliations:** 1 Laboratory of Inland Fisheries and Aquaculture, Department of Zoology, Faculty of Veterinary Medicine and Animal Science, Poznan University of Life Sciences, Wojska Polskiego 71c, 60-625 Poznan, Poland; joanna.kowalska@up.poznan.pl (J.K.); mateusz.rawski@up.poznan.pl (M.R.); jan.mazurkiewicz@up.poznan.pl (J.M.); 2 Experimental Station of Feed Production Technology and Aquaculture, Poznan University of Life Sciences, Muchocin 20, 64-400 Miedzychod, Poland; 3 Department of Animal Physiology, Physiotherapy and Nutrition, Faculty of Animal Breeding and Biology, Bydgoszcz University of Technology, Mazowiecka 28, 85-796 Bydgoszcz, Poland; bogucka@pbs.edu.pl (J.B.); e.ziolkowska@pbs.edu.pl (E.Z.); 4 Department of Animal Nutrition, Faculty of Veterinary Medicine and Animal Science, Poznan University of Life Sciences, Wołyńska 33, 60-637 Poznan, Poland; bartosz.kieronczyk@up.poznan.pl

**Keywords:** fish nutrition, rheophilic cyprinid fish, insect meals, nutriphysiology, feed utilization

## Abstract

**Simple Summary:**

The success of wild fish conservation strongly depends on the development of effective methods for restocking material rearing. An essential element in this process is the nutrition of juvenile stages of fish in controlled conditions, requiring the optimization of diet composition to meet specific nutritional and behavioral requirements. This study aimed to evaluate the possibility of using insect-derived meals as an alternative to fish meal in diets for the ide (*Leuciscus idus*) juveniles. Insects are a significant part of omnivorous fish diets, such as the ide natural diet, so their usage in aquaculture is strongly justified. Insect larval meals of two insect species, namely, black soldier fly and mealworm, improved the growth, feed utilization, and health of ide juveniles. However, superworm larval meal negatively affected the abovementioned parameters and is not recommended for the diet of juvenile cyprinid fish.

**Abstract:**

The ide (*Leuciscus idus*) is a native European species of rheophilic cyprinid fish whose wild population status is dependent on conservation efforts, particularly regular restocking. This study aimed to evaluate the effects of including insect meals as a component in the diet of ide juveniles on their growth performance, feed utilization, and nutriphysiological status. Four diets were formulated: three with insect meals, HI–with 20% *Hermetia illucens* meal, TM–with 20% *Tenebrio molitor* meal, and ZM–with 20% *Zophobas morio* meal, and the control group diet, CON–fish meal with no insect component. The effects of the various diets on the efficiency of rearing ide juveniles were assessed based on fish growth parameters, feed utilization parameters, somatic indices, and intestinal and hepatopancreatic histomorphology. The highest increase in fish weight gain and the protein efficiency ratio was observed in the HI and TM groups, while the lowest values were observed in the CON and ZM groups. Comparable results were noted for the feed conversion ratio, which was most favorable in the HI and TM groups and increased in the ZM group. The use of black soldier fly and mealworm larval meal in the diets of ide juveniles had a positive effect on rearing results and overall fish health.

## 1. Introduction

Freshwater ecosystems are threatened by continuously increasing human influence, and according to the IUCN Red List, approximately 31% of classified freshwater fish species are endangered, and for many more, data are deficient [1]. For these reasons, various conservation actions are being implemented around the world. These include management practices, fishery laws and fishing regulations, restocking and relocation, artificial breeding, and the creation of conservation reserves [2].

The ide (*Leuciscus idus*) is native Eurasian rheophilic cyprinid fish that inhabits mainly lowland rivers and lakes and is characterized by the ability to use a variety of fresh and brackish water habitats [3,4] and by a broad spectrum of food sources including mollusks, crustaceans, macrophytes, algae, detritus, and insects and their larvae [5]. Although ide is not a major species in fish production, it is sometimes reared in polyculture systems with common carp, especially in the case of extensive aquaculture and organic production in central European countries [6,7]. Its economic importance lies mainly as an ornamental and recreational angling fish species [5,8].

The IUCN Red List classifies ide as a species of least concern (LC) globally and in Europe. However, in some European countries, it is locally classified as “vulnerable” or “endangered” [5]. Among other rheophilic fish species, it is threatened by various human-originated factors, such as modifications in the natural flow of rivers, habitat changes, and water pollution [9]. Interestingly, the presence of ide (more so than other rheophilic fishes) in a given environment translates almost directly into increased biodiversity of the entire flowing-water ecosystem [10].

In the case of rheophilic cyprinid fish species, the biotechnologies for breeding, reproduction, and most aspects of rearing larval stages are well described and have been, or are currently, the subject of research and development studies [11,12,13]. Nevertheless, no best practices and practical diets have been designed specifically for this species, especially for growing juvenile stages. It can result in high variability of the quality of stocking juveniles produced in this way and thereby reduce the chances of adaptation in the environment [14]. To maintain natural biodiversity and allow recreational angling of ide, stocking material production and annual restocking actions are implemented in Poland. The production of ide for conservation purposes mainly occurs in recirculation aquaculture systems (RAS) with controlled conditions and reaches 5.2 million individuals annually, of which 89% are juveniles [15]. Thus, developing a standardized ide production procedure in controlled conditions seems highly recommended to produce the best quality stock for release. The most recent trend in aquaculture production is to increase its sustainability [16]. Many studies have considered novel feed components such as insect meals of various insect species as fish meal replacements and alternative protein sources; however, most of them focused on fish species farmed for human consumption [17,18]. In cyprinid fish aquaculture, several studies have conducted mainly on the use of black soldier fly (*Hermetia illucens*) and mealworm (*Tenebrio molitor*) in the nutrition of common carp (*Cyprinus carpio*) and its varieties [19,20,21], and grass carp (*Ctenopharyngodon idellus*) [22]. There are no data regarding insect meals in the diets of rheophilic cyprinid fish such as ide. As a result of those actions, some of the insect species, including the black soldier fly and mealworm, have been approved by the European Union for use in feeds for the aquaculture sector [23]. As of now, superworm (*Zophobas morio*) is not included in this law. Insects are among the most promising alternative protein sources for improving aquacultural fish nutrition [17,24]. It is due to insects having a high protein content, short life cycle, and ease of production [25]. They can also be a source of various bioactive compounds, including antimicrobial peptides, lauric acid, and chitin [26,27]. The fact that ide is an omnivorous species for which insects at various stages of development are an essential, natural food source, especially for juvenile fish, presents an unparalleled opportunity for developing an insect-derived diet for aquaculture.

Therefore, this study aimed to evaluate the effectiveness of insect meals in the diet of juvenile ide, focusing specifically on fish growth performance, gastrointestinal tract development, and overall fish health, and to develop feeds best suited for practical application in rearing the highest quality ide juveniles to increase restocking success.

## 2. Materials and Methods

### 2.1. Insect Meals and Diet Preparation

The *Hermetia illucens* meal was obtained from HiProMine S.A., Robakowo, Poland.

The substrates fed to the larvae were characterized by 22% dry matter content and were free from any animal products according to the EC regulation (No. 1069/09). The larvae were harvested at the 10th day of growth (prepupal stage), then sieved and washed on a drum separator for 10 min at 90 °C (HPM cleaning system, Robakowo, Poland). Next, the larvae were dried for 1h at 130 °C, then for 23 h at 80 °C to obtain constant dry matter with a chamber airflow dryer (HiProMine S.A., Poznań, Poland). After that, the larvae were ground to obtain insect meal.

Non-standardized fresh insect larvae of *Tenebrio molitor* and *Zophobas morio* were acquired from local breeders, frozen, then air-dried for 24 h at 50 °C to obtain constant dry matter, and ground to obtain insect meals. All insect meals were stored at 4 °C before use in diet preparation. Chemical analyses of insect meals were performed prior to diet formulations. The dry matter, crude protein, crude fat, crude fiber, crude ash, calcium, phosphorus content, and amino acid and fatty acid profiles were analyzed in the accredited laboratory J.S Hamilton Sp. z o.o. (Gdynia, Poland). Samples were analyzed according to the following norms and regulations: PB-116 ed. III of 11 August 2020 for crude protein, PN-EN ISO 6865:2002 for crude fiber, ISO 5984:2002 for crude ash, Commission Regulation (EC) No. 152/2009 of 27 January 2009, Annex III, Part A for dry matter, Commission Regulation (EC) No. 152/2009, Annex III, Part H, Methodology B for crude fat, PB-223/ICP, ed. II of 12 January 2015 for phosphorus, PB-223/ICP, ed. II of 12 January 2015 for calcium, PN-EN ISO 12966-1:2015-01, PN-EN ISO 12966-2:2017-05 except for points 5.3 and 5.5, PN-EN ISO 12966-4:2015-07 for fatty acid profile (detection threshold 0.5 g 100 g fat^−1^), and PB-53/HPLC ed. II of 30 December 2008 for amino acid profile.

Nitrogen-free extract content was calculated.

For chitin estimation and protein overestimation correction, the crude protein content of the prepared insect meals was estimated based on the amino acid composition [28,29]. The protein conversion factor (Kp) was applied and calculated according to the following formula established by Janssen et al. (2017) and Rawski et al. (2020) [24,30]:

Kp = (total amino acid content/total protein content based on N × 6.25 analysis) × 6.25(1)

All of the results of the chemical analysis of fish meal and our prepared insect meals are presented in Table 1.

The control diet (CON) was formulated with 300 g of fish meal per kilogram and no insect meal. Three experimental diets were formulated with a 50% replacement of fish meal by insect meals: diet HI—with 150 g of fish meal and 200 g of *Hermetia illucens* meal per kilogram, diet TM—with 150 g of fish meal and 200 g of *Tenebrio molitor* meal per kilogram, and diet ZM—with 150 g of fish meal and 200 g of *Zophobas morio* meal per kilogram (Table 2). The levels of nutrients in the diets were based on Ren et al. (2017) and dietary recommendations for cyprinids [31,32,33]. The differences in the amount of fish oil in diets were due to the various fat contents of insect meals (the highest in *Zophobas morio* meal and the lowest in *Hermetia illucens* meal). To maintain the same level of crude fat in all of the experimental diets, different amounts of fish oil, or no fish oil in the case of ZM diet, were needed.

**Figure 1:**
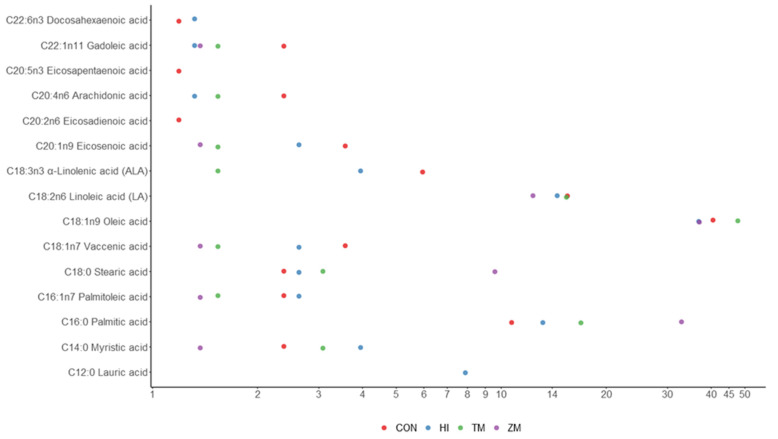
Fatty acids represented in more than 0.5% of lipids in experimental feeds for ide juveniles (the scale in the figure is logarithmic). CON—diet with 300 g of fish meal per kilogram and no insect meal; HI—diet with 150 g of fish meal and 200 g of *Hermetia illucens* meal per kilogram; TM—diet with 150 g of fish meal and 200 g of *Tenebrio molitor* meal per kilogram; ZM—diet with 150 g of fish meal and 200 g of *Zophobas morio* meal per kilogram.

**Figure 2:**
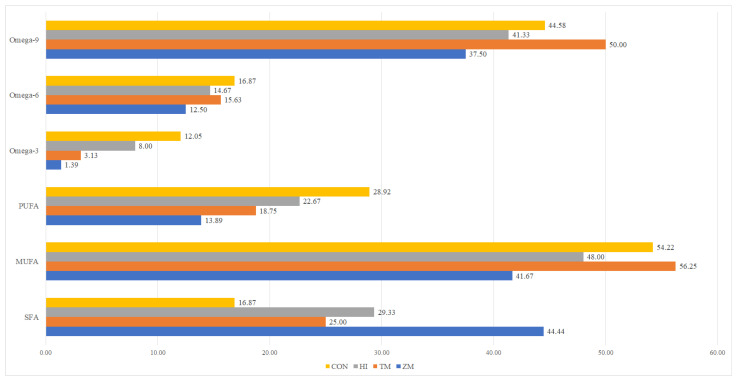
Composition of fatty acid groups (%) of lipids in experimental diets for ide juveniles. SFA—saturated fatty acids; MUFA—monounsaturated fatty acids; PUFA—polyunsaturated fatty acids. CON—diet with 300 g of fish meal per kilogram and no insect meal; HI—diet with 150 g of fish meal and 200 g of *Hermetia illucens* meal per kilogram; TM—diet with 150 g of fish meal and 200 g of *Tenebrio molitor* meal per kilogram; ZM—diet with 150 g of fish meal and 200 g of *Zophobas morio* meal per kilogram.

Chemical composition analysis showed that experimental diets formulated as isoenergetic (18.48–19.05 MJ kg^−1^) and with the same level of protein (42.3–44.7%) met the mentioned criteria (Table 3). Slight differences in protein levels were due to the formulation using Kp calculated for each of the insect meals used. Differences in crude fat levels were due to the different lipid contents of the insect larvae used to make the respective meals. There were no differences in gross energy levels and energy/protein ratios of formulated diets despite the mentioned factor variations. In the HI diet, a relatively high level of lauric acid was observed (Figure 1). Insect inclusion in the diet resulted in a decrease in omega-3, omega-6 and polyunsaturated fatty acids and an increase in saturated fatty acids (Figure 2). 

The gross energy of the diets was calculated from the chemical composition using the conversion factors for fish: carbohydrates, 17.2; protein, 23.6; and fat, 39.5 kJ g^−1^ [34].

Diets were prepared by extrusion processing with a twin-screw warm extruder at the Experimental Station of Feed Production Technology and Aquaculture in Muchocin (Poznan University of Life Sciences). The temperature of the extruder’s cylinder was 90 °C in the zone of increasing pressure and 120 °C in the zone of high pressure, and the head temperature was 130 °C. The screw speed was 52 rpm, while diameter of the used nozzle was 3mm. In postproduction, a vacuum coating with fish oil was applied (Rollermac BA 15 FR at. Pomati Group S.R. L, Codogno, Italy). The chemical compositions of the feeds were analyzed using the methods described above, and the results are shown in Table 3.

The diets were formulated to be consistently isoenergetic and to have the same crude protein level. Differences in crude fat levels and the amount of fish oil used were present due to the different lipid levels in the insect meals. The chemical composition and fatty acid profiles of the experimental diets were analyzed in one sample per treatment and are shown in Table 3, Figure 1 and Figure 2, and the Supplementary Materials (Tables S1 and S2).

### 2.2. Animal Husbandry and Growth Trial

Fish were kept in an experimental recirculation aquaculture system in 20 growth tanks, each with 400 dm^3^ net capacity. Stable environmental conditions (water temperature: 23 °C; photoperiod: 14 h of light and 10 h of darkness) were maintained during the growth trial. A total of 4000 ide juveniles with an average weight of 5 g were randomly divided into four groups, with five replicates each (200 fish/tank). The growth trial lasted 60 days. A feed dose was given to the fish according to their body weight and consumption on the previous days. Fish were bulk weighed every 10 days to adjust the dose of feed (days 0, 10, 20, 30, 40, 50, 60). During the entire experimental period, the mean daily water temperature was 22.5 °C, the mean level of oxygen dissolved in water was 7.68 mg O^2^/dm^3^ (WTW Multi Line P4 with an optical oxygen sensor, WTW, FDO 924, 99, Weilheim, Germany), the mean pH of water was 7.90 (WTW Multi Line P3 pH meter, WTW, Weilheim, Germany), the mean conductivity of water was 784 µS (HM Digital Inc., EC-3, Redondo Beach, CA, USA), the mean content of ammonia was 0.02 mg/dm^3^, the mean content of NO_2_ was 0.15 mg/dm^3^, and the mean content of NO_3_ was 14.5 mg/dm^3^ (Merck MColortest cat no. 1144230002, 1144240001, and 111170.0001, Merck Darmstadt, Germany). The following growth performance and dietary utilization parameters were calculated (n = 5): body weight gain (BWG), final body weight (FBW), percent body weight gain (PWG), specific growth rate (SGR), feed intake (FI), protein efficiency ratio (PER), feed conversion ratio (FCR), and survival rate (SR). Formulas that were used are given below:

FBW (g) = fish biomass in the tank (g)/number of fish in the tank(2)

BWG (g) = final body weight (g) − initial body weight (g)(3)

SGR (%/day) = ((ln final body weight − ln initial body weight)/number of feeding days) × 100(4)

PWG (%) = (final body weight (g) − initial body weight (g)/initial body weight (g)) × 100 (5)

FI (g) = applied feed (g) − uneaten feed (g)(6)

FCR = feed intake (g)/body weight gain (g)(7)

PER = (body weight gain (g)/(feed intake (g) × protein level in the diet (%))(8)

SR (%) = (final number of live fish/initial number of live fish) × 100(9)

### 2.3. Sampling

At the end of the growth trial (day 60), eight fish from each tank were euthanized using immersion in 500 mg/L of MS-222 solution and then dissected (n = 40). They were weighed for somatic indices calculation, and total fish length, length of the gastrointestinal tract, and viscera weight were measured (n = 40). For histological analyses, samples of the intestine (approximately 2 cm long) and hepatopancreas of three fish from each tank (n = 15) were taken postmortem directly and immersed in Bounin’s solution. For chemical composition analysis, 5 × 25 fish per tank were pooled and analyzed in one sample per treatment.

### 2.4. Somatic Indices

Somatic indices for evaluating the effect of diet on the internal organs and the fish condition were analyzed (n = 40) according to the methodology described by Piccolo et al. (2017) [35]. The indices include CF—condition factor, VSI—viscerosomatic index, and RGL—relative gastrointestinal tract length. They were calculated according to the formulas presented below:

CF = (body weight (g)/fish total length (cm)^3^) × 100 (10)

VSI (%) = (viscera weight (g)/body weight (g)) × 100(11)

RGL (%) = (gastrointestinal tract length (mm)/fish total length (mm)) × 100(12)

### 2.5. Histological Analyses

Segments of the intestine (n = 15) were individually rinsed with 0.9% normal saline and preserved in 4% formalin, which was buffered with CaCO_3_ solution. Samples preserved in this way were then cleared, dehydrated, and infiltrated with paraffin using a tissue processor (Thermo Shandon, Runcorn, UK). Then, the samples were embedded in paraffin blocks in a paraffin embedding system (Medite, Burgdorf, Germany). Rotatory microtome (Thermo Shandon, Runcorn, UK) was used to cut the paraffin blocks into 10 µm slices. The slices were coated in chicken egg whites (with added glycerin) and placed on microscope slides. Then, the paraffin was removed from samples, which were rehydrated and stained with PAS (Periodic acid-Schiff) technique for the analysis of intestinal morphometry. To measure the thickness of the muscular layer as well as width and height of villi, a microscope (Nikon Ci-L) with integrated camera (Nikon DS-Fi3) and NIS Elements software (Nikon Instruments Inc., Tokyo, Japan) was used. For the measurement of the height of the villi, 10 samples were selected randomly on the cross-section of a sample. The length of the villi was measured from the top to the base of the villus. The measurements of the width of the villi were taken at the midpoint of villus length. The calculations of the surface area of the villi were based on the formula described by Sakamoto et al. (2000):

(2π) × (VW/2) × (VH)(13)

where VW = villus width and VH = villus height [36].

Histological examination of the hepatopancreas samples (n = 15) was performed based on the paraffin method and hematoxylin and eosin staining. After preparing the slides, 10 images per sample of the liver were used for the analysis of qualitative and quantitative data. A semiquantitative scoring system was used to evaluate the severity of histopathological changes in the hepatopancreas of ide in the presence of vacuoles in hepatocytes and characteristic fat vacuolization, the number of congestions, necrosis, and fibrosis. The protocol provided by Peebua et al. (2006) and Elia et al. (2018) was used with some modifications [37,38]. The scoring system included a 5-point (0–4) scale in which 0 represented no changes, 1 represented slight histopathology present in less than 25% of fields, 2 represented mild histopathology present in less than 50% of fields, 3 represented moderate histopathology present in less than 75% of fields, and 4 represented severe histopathology observed in more than 75% of fields.

### 2.6. Statistical Analyses

Statistical calculations were performed using R software (version 4.0.3). The normality assessment of data was performed using the Shapiro–Wilk test, and the homogeneity of variance was confirmed using the Levene test. The occurrence of significant differences between the groups was evaluated using one-way analysis of variance (ANOVA), and for multiple comparisons, Duncan’s test was used for parameters with a normal distribution of data. The level of significance was determined at *p* ≤ 0.05. In the case of a lack of normality of data distribution, significant differences between groups were confirmed with a Kruskal–Wallis test. For multiple comparisons, Dunn’s test with the Benjamini–Hochberg method of controlling the false discovery rate was used. The level of significance was also determined at *p* ≤ 0.05.

### 2.7. Ethics Statement

All protocols, methods, and animal handling complied with the European Union’s recommendations (Directive 2010/63/EU) as well as the Polish law of 15 January 2015 on the protection of animals used for scientific purposes, the best practices and recommendations of the National Ethics Committee for Animal Experiments, and the Local Ethics Committee for Animal Experiments of Poznan University of Life Sciences. All personnel involved and in contact with the animals were trained in theory and practical applications of animal care, welfare, and experimental procedures by the Polish Laboratory Animal Science Association (certificates nr 5681/2021, 5580/2021, 5588/2021). All procedures and experiments complied with the guidelines, and all efforts were made to minimize animal suffering.

## 3. Results

### 3.1. Growth Performance and Feed Utilization

There was no fish mortality during the entire test period. The highest PWG and SGR were observed in the HI and TM groups, while the lowest were in the CON and ZM groups. The HI and TM groups were characterized by the lowest FCR and highest PER, while the ZM group had the most increased FCR and lowest PER. The control group was also characterized by the lowest PER, but the FCR was not different from that of the other groups (Table 4).

### 3.2. Condition and Somatic Indices

The ZM group lowered the condition factor (CF) of fish in comparison to the control and TM groups, and no statistically significant differences were observed between the ZM and HI treatments or among the CON, HI, and TM groups. The viscerosomatic index (VSI) was decreased by the HI and ZM diets compared to the control treatment. There were no differences between CON and TM or HI and TM. The significantly lowest value was observed in the ZM treatment. None of the tested diets affected fish gastrointestinal tract length (Table 5).

### 3.3. Chemical Composition of Fish

**Figure 3:**
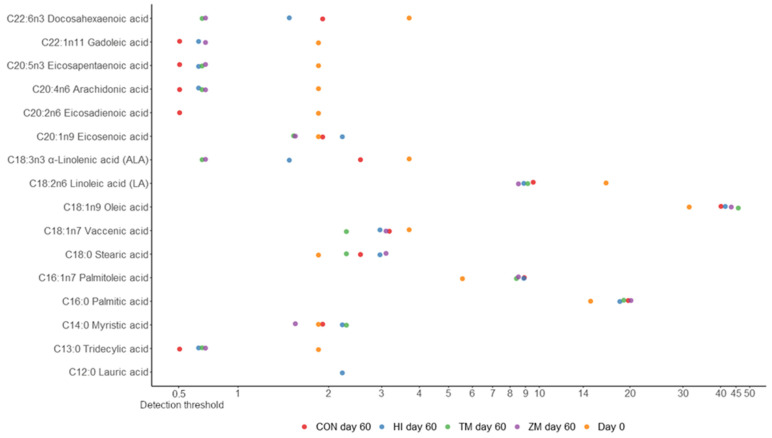
Fatty acids represented in more than 0.5% of lipids in fish samples of ide juveniles (the scale in the figure is logarithmic). CON—diet with 300 g of fish meal per kilogram and no insect meal; HI—diet with 150 g of fish meal and 200 g of *Hermetia illucens* meal per kilogram; TM—diet with 150 g of fish meal and 200 g of *Tenebrio molitor* meal per kilogram; ZM—diet with 150 g of fish meal and 200 g of *Zophobas morio* meal per kilogram.

**Figure 4:**
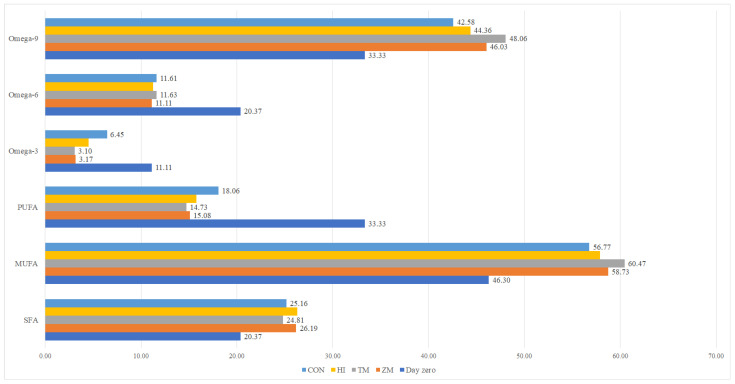
Composition of fatty acid groups (%) of lipids in ide samples on day zero and at the end of the experiment. SFA—saturated fatty acids; MUFA—monounsaturated fatty acids; PUFA—polyunsaturated fatty acids. CON—diet with 300 g of fish meal per kilogram and no insect meal; HI—diet with 150 g of fish meal and 200 g of *Hermetia illucens* meal per kilogram; TM—diet with 150 g of fish meal and 200 g of *Tenebrio molitor* meal per kilogram; ZM—diet with 150 g of fish meal and 200 g of *Zophobas morio* meal per kilogram; Day zero—a sample taken from all fish before allocating them to experimental groups.

Fish from all experimental groups were characterized by similar basic chemical compositions (Table 6) and fatty acid profiles (Figure 3 and Figure 4). However, some numerical value changes could be observed compared to samples taken before the experiment (lower omega-3, omega-6 fatty, and PUFA acid contents; higher SFA, MUFA, and omega-9 fatty acid contents; and higher fat contents).

### 3.4. Histopathology of the Hepatopancreas and Histomorphology of Intestine

In histopathological analyses of the hepatopancreas, differences were observed only in fat vacuolization and fibrosis. The highest number of fat vacuoles occurred in the TM group, while the lowest number occurred in the CON and ZM groups. In the case of fibrosis, the highest occurrence was noted in the TM group, while the lowest was in the CON group. The HI and TM groups were not significantly different from the other groups. It should be noted that in all experimental groups, severe pathological changes were not observed, only mild changes (up to 2 points on a 5-point scale). Detailed data are given in Table 7. 

Significant differences occurred between the TM and ZM groups in terms of intestinal villi height and villi surface area. The TM group was characterized by increased villi height and surface area compared to the ZM group. For the CON and HI treatments, no significant differences were found in comparison to the rest of the groups (Table 8).

## 4. Discussion

Ide is a poorly studied and understood species [5]. This study is the first case of insect meal application in *Leuciscus idus* juvenile nutrition. Moreover, in the widely available scientific literature, only one study aimed to find the optimal protein content in the diet for ide [31]; the other studies investigated different fish species from the same family, Cyprinidae, mainly common carp (*Cyprinus carpio*). Fish dietary requirements are species-specific and depend on age, physiological state, and many other factors [34]. The best growth performance results for ide were obtained with 36.9–37.7% crude protein content in dry matter of feed, and dietary methionine and lysine levels were calculated as 0.83% and 2.25% of the dry matter of feed, respectively, to ensure optimal growth of juveniles [31].

In animal nutrition, the nitrogen to protein conversion factor (Kp) is widely assumed to be N × 6.25 due to the average content of 16 g of nitrogen per 100 g of protein. In the case of insect meals used as a protein source in this experiment, N was calculated based on amino acid content and varied from 4.91 to 5.77, which agrees with previous studies, confirming the overestimation of crude protein content using the N × 6.25 calculation [24,28]. For the first time in the scientific literature, we assessed the dietary effects of including insect meals of three different insect species in rheophilic cyprinid fish aquaculture. *Zophobas morio* meal amino acid-based Kp application showed 4.92% overestimation compared to the N × 6.25 calculation; for *Tenebrio molitor* and *Hermetia illucens* meals, the overestimation was 22.07 and 15.93%, respectively. However, applying Kp in whole-feed composition analysis is impossible due to the lack of analytic differentiation of nitrogen of insect and non-insect origin. For these reasons, a slight increase in crude protein level was observed in the diets containing insect meal, which was caused by non-amino acid nitrogen bound in chitin. This kind of slight miscalculation was present even when Kp was used. In order to avoid greater overestimation of crude protein content, we suggest balancing practical insect-containing feeds using amino acid content and Kp to ensure that the nutritional requirements of fish are fully met.

The effective utilization of nutrients is closely linked to an appropriately balanced energy level, which, in practice, is most frequently expressed as the energy/protein ratio [39]. The diet for fish should contain from 33.5 to 42 kJ of energy per 1 g of total protein since this guarantees that the dietary protein will be used for fish growth and not only to cover the requirements for energy [39,40]. 

As cold-blooded animals, fish have far lower energy requirements than warm-blooded animals. Chakraborty et al. (1992) and Kaushik (1995) demonstrated a linear correlation between nitrogen retention and the energy level in the diet [41,42]. Diets with energy levels that are too low result in increased individual protein utilization as an energy source, reduced growth performance, and worsened feed utilization. The energy/protein ratio in the experimental diets met those recommendations and oscillated from 43.90 to 44.91 kJ gross energy per 1 g of crude protein. The favorable feed conversion ratio (FCR) and body weight gain confirmed that the energy levels in the experimental diets were well balanced.

In omnivorous fish nutrition, fat and carbohydrates are the dietary components that effectively cover energy requirements, which is why the energy level in the diet is more important than the quantity of fat. Takeuchi et al. (1979b) demonstrated that an increase in the amount of digestible energy in the diet from 13 to 15 MJ kg^−1^ with a 5–15% fat addition did not result in better carp growth or higher protein retention [43]. The crude fat levels in the diets applied in this study varied due to the quite different fat content of the various insect meals. Due to the facts mentioned above, all experimental diets were balanced according to the energy/protein ratio. Ide, as a species with no history of domestication or selection aimed at increasing fish production, is characterized by slower and less intensive growth than common carp; thus, its energetic requirements have not been empirically determined. However, it was shown that the application of low-fat diets (6.5–8.4% fat level) resulted in increased body fat accumulation. This proves that common carp nutritional requirements (12% fat level) should not be applied for ide as a simple extrapolation [42]. Even though the fat level in the fish body was more than doubled during the experiment in the present study, it was observed that in the HI, TM, and ZM groups, the fat level was 14.01, 16.56, and 17.83% lower, respectively, than in the control treatment (Table 6).

Dietary fats should not be considered as an energy source only. They contain fatty acids crucial for fish metabolism. To date, the requirements of common carp have been determined for only two fatty acids, namely, linoleic (18:2 n-6) and linolenic (18:3 n-3) acid, whose levels in dietary lipids should be more than 1% each [44]. The amount of these fatty acids in diets formulated for ide corresponded to those recommendations and was on the level of 12.33 to 15.48 for linoleic and <0.5 to 5.95 for linolenic acid. However, only with the ZM diet did the replacement of fish meal with *Zophobas morio* meal result in a linolenic acid level lower than 1% dietary fat. We observed that polyunsaturated fatty acids (PUFAs) and omega-3 fatty acids decreased in all insect-containing diets. At the same time, it is worth noting that, in the case of the HI diet, the amount of beneficial lauric acid (C12:0) increased, and the amount of eicosadienoic acid (C20:2n6) was on the same level as in the control (detailed values are presented in Tables S1 and S2). Similar results were observed by Zhou et al. (2018) for Jian carp (*Cyprinus carpio* var. Jian) [21]. Thus, we suggest enriching the diet of insect larvae with highly unsaturated fatty acids before processing them into insect meal to prevent the decrease in PUFA and omega-3 fatty acids in fish.

Ogino et al. (1976) and Takeuchi et al. (1979a) found that carbohydrates can be used as primary energy sources in the common carp diet and indicated that the optimum range of carbohydrates in the feed for carp is from 30 to 40% [40,43]. The high effectiveness of carbohydrate utilization (mainly starch) as a source of energy for common carp stems from the activity of amylolytic enzymes, which is higher than that in predatory fish species [45]. It is justified to assume that these characteristics seem similar for ide; thus, experimental diets were formulated with 40.01 to 42.33% carbohydrates. Additionally, preparation of carp feed with the extrusion method significantly improves the starch’s assimilability, which increases the digestible energy content of the diet [46].

Another nutrient that should be considered when formulating insect-containing diets is crude fiber. Insect meal dietary inclusion increases crude fiber content due to exoskeletons of larvae made from chitin, which is considered a fiber-like material. In the case of an omnivorous fish, a slight increase in crude fiber does not negatively influence growth performance and digestion processes [47,48].

To date, there are no studies that have aimed to use insect meals in diets for ide (*Leuciscus idus*). However, studies have been conducted on live invertebrate application for the early life stages of ide, assessing natural food (mainly *Artemia salina*) and composed feed for common carp [8,49]. The studies’ findings agree that for ide larvae, providing live food results in an increased survival rate compared to extruded dry feeds, thus being more effective and profitable [50]. Nevertheless, live invertebrate application can be utilized only in the first stage of fish rearing due to its unbalanced nutritional value and possible contamination with pathogens and heavy metals [51,52,53]. Insects are the primary natural food source for many fish species [5,54,55]. However, due to controlled rearing conditions and high hygienic standards of processing insect-derived products, insect meals are not affected by those disadvantages. Free choice feeding and other food acceptance tests have proven their high palatability and thus their potential application in the nutrition of farm [24] and ornamental [56] fish, including common carp [57].

The growth performance results for ide juveniles from all experimental groups except for ZM were satisfactory and comparable to those obtained by Ren et al. (2017), with similar dietary protein content in the case of feed conversion ratio (FCR) [31]. The specific growth rate (0.65–1.00) of ide juveniles in the present study was lower than that observed in common carp (1.12–3.12) depending on breeding technology and environmental and dietary factors [58,59,60]. The feed conversion ratio values for groups HI (2.02) and TM (2.01) were close to the average for carp [61]. It should be noted that ide is a non-domesticated fish, while common carp is a species that has been farmed and selectively bred for centuries. Histopathological changes, such as increased fibrosis and fat vacuolization in the hepatopancreas, occurred mainly in the TM group. These results contradict those obtained by Li et al. (2017) for Jian carp [20]. They reported tissue disruptions in the intestine with 7.9% and more defatted black soldier larvae meal (BSFM) in the fish diet, corresponding to at least 75% fish meal replacement and decreased lipid deposits in the hepatopancreas. However, this reduction in lipid deposits is probably connected to the specific fatty acid profiles of insect meals and the various overall amounts of insect fat in the diet. Contrary to the presented research, Gebremichael et al. (2021) found that replacement of fish meal with BSFM (up to 100%) in the common carp diet did not affect the growth performance, feed utilization, relative gastrointestinal tract length, or hepatosomatic index of the fish [19]. Significant changes occurred only in the viscerosomatic index (VSI), decreasing along with the increased amount of BSFM in the diet. We also noted lower VSI in the HI group than in the control. The overall worsening of ide growth in the ZM group might be connected to the diet’s highest insect fat, resulting in no fish oil inclusion and some nutritional deficiencies of essential fatty acids. Further studies are needed to determine the exact effects of replacement of fish oil with *Zophobas morio* fat in the diets for ide.

The dietary insect meal mode of action should be discussed not only at the level of growth performance but also based on its nutriphysiological effects. Experimental diets (HI, TM) positively affected the physiology of the intestine by increasing villi height and the overall surface of nutrient digestion and absorption, which can be connected to better growth performance [62]. Histological changes probably connected to the high fat content of insect meals occurred in the hepatopancreas, although they were not severe or biologically significant. Moreover, insect meal inclusion did not cause a higher accumulation of fat reserves in fish bodies than in the control group. During the experiment, no fish mortality was observed, and condition and somatic indices were not negatively affected, so it can be assumed that the ide juveniles were in optimal health.

The species-specific innovative diets for ide juveniles used in this study have proven to be effective and potentially beneficial for cyprinid fish. Nevertheless, further studies are required to optimize diet formulation and determine optimal insect meal usage. The current publication’s novelties open new perspectives in the area of insect meal application in aquaculture and rheophilic fish conservation. For the first time, the high usability of a diet containing insect meal was proven beyond use in widely farmed, domesticated species to include successful application in culturing juvenile cyprinids raised for restocking purposes.

## 5. Conclusions

The use of 20% dietary inclusion of insect meals derived from *Hermetia illucens* or *Tenebrio molitor* larvae in ide juveniles’ nutrition was proven to positively affect both growth performance and feed utilization parameters. We also observed better fish nutriphysiological status in terms of gastrointestinal tract development in the HI and TM groups than in the control group treatment. It should also be noted that the use of *Zophobas morio* larvae meal is not recommended due to the worsening of growth and overall health of fish fed on this diet.

## Figures and Tables

**Table 1:** Chemical and amino acid compositions of fish meal and insect meals used for preparing experimental diets.

Nutrient (% Fresh Matter)	Fish Meal	*Hermetia illucens* Meal	*Tenebrio molitor* Meal	*Zophobas morio* Meal
Dry matter	91.10	94.97	95.20	96.90
Crude fiber	3.40	5.70	7.30	5.50
Ash	8.82	8.08	4.10	2.83
Crude fat	9.00	12.06	26.60	37.40
Nitrogen-free extract	16.58	16.29	2.5	2.77
Crude protein (N × 6.25)	53.30	52.84	54.70	48.40
Kp	6.25	5.12	4.91	5.77
The crude protein, according to Kp	53.30	45.58	44.81	46.13
Amino acids (g 100 g protein^−1^ N × 6.25)				
Aspartic acid	4.79	1.60	2.77	3.50
Glutamic acid	6.25	3.44	5.16	6.49
Serine	1.82	1.32	2.28	2.24
Glycine	2.04	1.75	2.77	2.58
Histidine	0.89	0.74	1.40	1.50
Arginine	2.66	1.32	2.37	2.45
Threonine	1.44	1.19	1.92	1.97
Alanine	1.87	3.01	4.41	3.88
Proline	2.03	1.98	3.32	2.94
Tyrosine	1.00	1.58	2.81	3.60
Valine	1.92	1.70	2.87	2.90
Methionine	0.11	0.49	0.65	0.60
Cystine	0.34	0.22	0.35	0.32
Isoleucine	1.76	1.25	2.08	2.15
Leucine	2.94	1.94	3.52	3.49
Phenylalanine	1.95	1.05	1.62	1.81
Lysine	2.25	1.36	2.36	2.28

Kp—nitrogen to protein conversion factor.

**Table 2:** The composition of the experimental diets for ide juveniles used in the experiment.

Ingredient (g kg^−1^)	Diets			
	CON	HI	TM	ZM
Fish meal	300	150	150	150
Red blood cells	90	90	90	90
Insect meal	0	200	200	200
Soy protein isolate	80	80	80	80
Wheat gluten	100	100	100	100
Wheat meal	125	125	125	125
Corn starch	196	155	186	201
Fish oil	61	50	15	0
Soybean lecithin	10	10	10	10
Premix ^1^	15	15	15	15
Vitamin premix ^2^	1	1	1	1
Choline chloride	2	2	2	2
Fodder chalk	20	22	26	26
Vitamin C ^3^	0.5	0.5	0.5	0.5

CON—diet with 300 g of fish meal per kilogram and no insect meal; HI—diet with 150 g of fish meal and 200 g of *Hermetia illucens* meal per kilogram; TM—diet with 150 g of fish meal and 200 g of *Tenebrio molitor* meal per kilogram; ZM—diet with 150 g of fish meal and 200 g of *Zophobas morio* meal per kilogram; ^1^ Premix–containing: vitamin D3 200,000 IU, vitamin A 1,000,000 IU, vitamin K 0.2 g, vitamin E 1.5 g, vitamin B1 0.05 g, vitamin B2 0.4 g, nicotinic acid 2.5 g, vitamin B12 0.001 g, D-calcium pantothenate 1.0 g, inositol 35 g, folic acid 0.1 g, choline chloride 7.5 g, methionine 150.0 g, lysine 150.0 g, Mn 6.5 g, Fe 2.5 g, Cu 0.8 g, Zn 4.0 g, Co 0.04 g, and J 0.008 g per 1 kg; ^2^ Vitazol AD3EC, BIOWET Drwalew, Poland–containing: vitamin D3 5000 IU, vitamin A 50,000 IU, vitamin C 100.0 mg, vitamin E 30.0 mg per 1 kg; ^3^ Stay C, DSM Nutritional Products Ltd., Mszczonów, Poland.

**Table 3:** Analyzed chemical composition, amino acid profile, and energetic value of experimental feeds for ide juveniles.

Nutrient (% Fresh Matter)	Diets			
	CON	HI	TM	ZM
Dry matter	91.2	91.0	91.8	91.6
Crude protein	42.3	44.0	44.7	43.5
Amino acids (g 100 g protein^−1^)				
Aspartic acid	6.52	6.25	6.29	6.16
Glutamic acid	16.24	15.48	15.55	15.66
Serine	4.23	4.32	4.41	4.23
Glycine	3.57	3.82	3.94	3.72
Histidine	2.65	2.75	2.77	2.71
Arginine	4.14	4.07	4.16	4.02
Threonine	2.86	3.05	3.06	2.99
Alanine	4.21	4.98	5.23	4.83
Proline	5.60	5.98	6.20	5.93
Tyrosine	2.22	2.86	2.75	2.99
Valine	3.90	4.14	4.18	4.09
Methionine	1.42	1.59	1.57	1.54
Cystine	0.66	0.64	0.65	0.60
Isoleucine	2.20	2.32	2.35	2.32
Leucine	6.97	7.00	7.23	7.10
Phenylalanine	4.44	4.41	4.32	4.34
Lysine	4.99	4.95	5.17	5.06
Crude fat	8.3	7.5	6.4	7.2
Ash	5.57	5.99	5.73	5.38
Crude fiber	1.4	2.4	2.1	1.6
Nitrogen-free extract	42.33	40.01	40.97	42.22
Calcium	1.24	1.20	1.28	1.27
Phosphorus	0.51	0.59	0.57	0.49
Gross energy (MJ kg^−1^)	18.77	18.57	18.48	19.05
Energy/protein ratio (kJ g^−1^ protein)	44.47	43.90	44.43	44.91

CON—diet with 300 g of fish meal per kilogram and no insect meal; HI—diet with 150 g of fish meal and 200 g of *Hermetia illucens* meal per kilogram; TM—diet with 150 g of fish meal and 200 g of *Tenebrio molitor* meal per kilogram; ZM—diet with 150 g of fish meal and 200 g of *Zophobas morio* meal per kilogram.

**Table 4:** Growth performance and feed utilization of ide fed experimental diets.

Parameter	Diets					
	CON	HI	TM	ZM	SEM	*p* Value
Final individual body weight (g^−1^)	6.94 ^b^ ± 0.37	7.88 ^a^ ± 0.38	7.99 ^a^ ± 0.25	6.59 ^b^ ± 0.50	0.1582	0.0000413
Mean individual body weight gain (g^−1^)	2.53 ^b^ ± 0.35	3.41 ^a^ ± 0.27	3.60 ^a^ ± 0.53	2.14 ^b^ ± 0.56	0.1658	0.000196
Specific growth rate (SGR, % day^−1^)	0.76 ^b^ ± 0.11	0.94 ^a^ ± 0.05	1.00 ^a^ ± 0.18	0.65 ^b^ ± 0.16	0.0423	0.00253
Percent weight gain (PWG, %)	57.9 ^b^ ± 9.8	76.4 ^a^ ± 5.1	83.3 ^a^ ± 19.0	48.5 ^b^ ± 14.4	4.1838	0.00238
Feed conversion ratio (FCR)	2.54 ^ab^ ± 0.39	2.02 ^b^ ± 0.14	2.01 ^b^ ± 0.35	3.08 ^a^ ± 0.87	0.1465	0.0127
Protein efficiency ratio (PER)	0.95 ^b^ ± 0.12	1.18 ^a^ ± 0.08	1.22 ^a^ ± 0.20	0.81 ^b^ ± 0.21	0.0512	0.00295

Results for each treatment are given as mean ± standard deviation. Differences between treatments (*p* ≤ 0.05) are indicated by different letters. CON—diet with 300 g of fish meal per kilogram and no insect meal; HI—diet with 150 g of fish meal and 200 g of Hermetia illucens meal per kilogram; TM—diet with 150 g of fish meal and 200 g of *Tenebrio molitor* meal per kilogram; ZM—diet with 150 g of fish meal and 200 g of Zophobas morio meal per kilogram.

**Table 5:** Condition and somatic indices of ide fed experimental diets.

Parameter	Diets					
	CON	HI	TM	ZM	SEM	*p* Value
CF	1.58 ^a^ ± 0.09	1.55 ^ab^ ± 0.08	1.57 ^a^ ± 0.08	1.51 ^b^ ± 0.11	0.0075	0.0043
VSI [%]	11.65 ^a^ ± 1.32	10.96 ^b^ ± 1.53	11.08 ^ab^ ± 1.32	10.15 ^c^ ± 1.48	0.1186	<0.0001
RGL [%]	104.02 ± 8.25	103.11 ± 6.27	102.89 ± 7.71	101.69 ± 10.51	0.6552	0.6600

Results for each treatment are given as mean ± standard deviation. Differences between treatments (*p* ≤ 0.05) are indicated by different letters. CON—diet with 300 g of fish meal per kilogram and no insect meal, HI—diet with 150 g of fish meal and 200 g of *Hermetia illucens* meal per kilogram; TM—diet with 150 g of fish meal and 200 g of *Tenebrio molitor* meal per kilogram; ZM—diet with 150 g of fish meal and 200 g of *Zophobas morio* meal per kilogram; CF—condition factor; VSI—viscerosomatic index; RGL—relative gastrointestinal tract length.

**Table 6:** Chemical composition of ide samples fed experimental diets.

% Fresh Matter	Day Zero	Groups on Day 60			
		CON	HI	TM	ZM
Dry matter	24.5	35.2	33.2	33.6	33.8
Crude protein	16.1	17.0	17.1	16.6	17.2
Crude fat	5.4	15.5	13.3	12.9	12.6
Ash	3.53	3.13	3.24	3.13	3.48
Calcium	1.06	0.74	0.96	0.87	1.06
Phosphorus	0.58	0.51	0.54	0.54	0.69

CON—diet with 300 g of fish meal per kilogram and no insect meal; HI—diet with 150 g of fish meal and 200 g of *Hermetia illucens* meal per kilogram; TM—diet with 150 g of fish meal and 200 g of *Tenebrio molitor* meal per kilogram; ZM—diet with 150 g of fish meal and 200 g of *Zophobas morio* meal per kilogram; Day zero—a sample taken from all fish before allocating them to experimental groups.

**Table 7:** Histological assessment of the hepatopancreas of ide juveniles fed experimental diets.

Parameters (0–4 Scale)	Groups					
	CON	HI	TM	ZM	SEM	*p* Value
Necrosis	0.27 ± 0.59	0.27 ± 0.46	0.47 ± 0.64	0.13 ± 0.35	0.0676	0.3758
Hepatocyte vacuolization	1.07 ± 0.96	1.93 ± 0.88	1.93 ± 1.03	1.47 ± 1.06	0.1328	0.0489
Congestion	1.13 ± 1.06	0.73 ± 0.80	0.53 ± 0.64	0.6 ± 0.51	0.1026	0.3529
Parenchymal eclipse	0.27 ± 0.46	0.4 ± 0.63	0.27 ± 0.70	0.07 ± 0.26	0.0698	0.2948
Fat vacuolization	0.8 ^a^ ± 1.0	1.6 ^ab^ ± 1.12	2.0 ^b^ ± 0.93	1.0^a^ ± 1.13	0.1462	0.0140
Fibrosis	0.0 ^a^ ± 0.0	0.07 ^ab^ ± 0.26	0.27 ^b^ ± 0.46	0.0^ab^ ± 0.0	0.0360	0.0264

Results for each treatment are given as mean ± standard deviation. Differences between treatments (*p* ≤ 0.05) are indicated by different letters. Assessment with use of a 5-point (0–4) scale in which 0 represents no changes, 1 represents slight histopathology present in less than 25% of fields, 2 represents mild histopathology present in less than 50% of fields, 3 represents moderate histopathology present in less than 75% of fields, and 4 represents severe histopathology observed in more than 75% of fields; CON—diet with 300 g of fish meal per kilogram and no insect meal; HI—diet with 150 g of fish meal and 200 g of *Hermetia illucens* meal per kilogram; TM—diet with 150 g of fish meal and 200 g of *Tenebrio molitor* meal per kilogram; ZM—diet with 150 g of fish meal and 200 g of *Zophobas morio* meal per kilogram.

**Table 8:** Histological parameters of the intestine of ide juveniles fed experimental diets.

Parameters	Groups					
	CON	HI	TM	ZM	SEM	*p* Value
Villi height (µm)	286.64 ^ab^ ± 74.82	283.77 ^ab^ ± 54.06	308.36 ^a^ ± 38.42	239.41 ^b^ ± 51.68	7.7931	0.0052
Villi width (µm)	87.31 ± 10.65	91.06 ± 12.61	92.02 ± 10.30	84.88 ± 9.41	1.4095	0.2470
Villi surface area (µm^2^)	79798 ^ab^ ± 29578	82202 ^ab^ ± 24301	89715 ^a^ ± 18839	64205 ^b^ ± 17622	3144.4	0.0117
Muscular layer thickness (µm)	36.06 ± 8.69	34.27 ± 8.35	34.54 ± 5.03	33.91 ± 5.35	0.8940	0.8470

Results for each treatment are given as mean ± standard deviation. Differences between treatments (*p* ≤ 0.05) are indicated by different letters. CON—diet with 300 g of fish meal per kilogram and no insect meal; HI—diet with 150 g of fish meal and 200 g of Hermetia illucens meal per kilogram; TM—diet with 150 g of fish meal and 200 g of *Tenebrio molitor* meal per kilogram; ZM—diet with 150 g of fish meal and 200 g of *Zophobas morio* meal per kilogram.

## Data Availability

The data presented in this study are available on request from the corresponding author.

## Supplementary Materials

The following are available online at https://www.mdpi.com/article/10.3390/ani12101227/s1, Table S1: Analyzed fatty acid profile of experimental feeds for ide juveniles (single analysis), Table S2: Analyzed fatty acid profile of ide samples fed experimental diets (single analysis).
