# Supplementary material for: Dietary Fish Meal Replacement with Hermetia illucens and Tenebrio molitor Larval Meals Improves the Growth Performance and Nutriphysiological Status of Ide (Leuciscus idus) Juveniles
Source: Animals (Basel). 2022 May 10;12(10):1227. doi: 10.3390/ani12101227 (PMC9137923; doi:10.3390/ani12101227)
Supplement: Supplementary file 1 [file animals-12-01227-s001.zip › animals-1691951-supplementary.pdf]

Table S1. Analyzed fatty acid profile of experimental feeds for ide juveniles.

Fatty acids [g 100g fat^{-1}]	Diets			
	CON	HI	TM	ZM
C4:0 Butyric acid	<0.5	<0.5	<0.5	<0.5
C6:0 Caproic acid	<0.5	<0.5	<0.5	<0.5
C8:0 Caprylic acid	<0.5	<0.5	<0.5	<0.5
C10:0 Capric acid	<0.5	<0.5	<0.5	<0.5
C11:0 Undecylic acid	<0.5	<0.5	<0.5	<0.5
C12:0 Lauric acid	<0.5	7.89	<0.5	<0.5
C13:0 Tridecylic acid	<0.5	<0.5	<0.5	<0.5
C14:0 Myristic acid	2.38	3.95	3.08	1.37
C14:1 Myristoleic acid	<0.5	<0.5	<0.5	<0.5
C15:0 Pentadecylic acid	<0.5	<0.5	<0.5	<0.5
C15:1 Ginkgolic	<0.5	<0.5	<0.5	<0.5
C16:0 Palmitic acid	10.71	13.16	16.92	32.88
C16:1n7 Palmitoleic acid	2.38	2.63	1.54	1.37
C16:1 total amount	2.38	2.63	1.54	1.37
C17:0 Margaric acid	<0.5	<0.5	<0.5	<0.5
C16:2n4 Hexadecadienoic acid	<0.5	<0.5	<0.5	<0.5
C17:1 Margaroleic acid	<0.5	<0.5	<0.5	<0.5
C16:3n4 Hexadecatrienoic acid	<0.5	<0.5	<0.5	<0.5
C18:0 Stearic acid	2.38	2.63	3.08	9.59
C18:1n9 Elaidic acid	<0.5	<0.5	<0.5	<0.5
C18:1n9 Oleic acid	40.48	36.84	47.69	36.99
C18:1n7 Vaccenic acid	3.57	2.63	1.54	1.37
C18:1 total amount	44.05	39.47	49.23	36.99
C18:2n6 Linoleic acid	<0.5	<0.5	<0.5	<0.5
C18:2 total trans amount	<0.5	<0.5	<0.5	<0.5
C18:2 total amount	15.48	14.47	15.38	12.33
C18:2n6 Linoleic acid (LA)	15.48	14.47	15.38	12.33
C20:0 Arachidic acid	<0.5	<0.5	<0.5	<0.5
C18:3n6 ɣ-Linolenic acid (GLA)	<0.5	<0.5	<0.5	<0.5
C21:0 Heneicosylic acid	<0.5	<0.5	<0.5	<0.5
C18:3n4 Octadecatrienoic acid	<0.5	<0.5	<0.5	<0.5
C20:1n9 Eicosenoic acid	3.57	2.63	1.54	1.37
C20:1 total amount	3.57	2.63	1.54	1.37
C18:3n3 α-Linolenic acid (ALA)	5.95	3.95	1.54	<0.5

CON – diet with 300 g of fish meal per kilogram and no insect meals inclusion, HI – diet with 150 g of fish meal and 200 g of *Hermetia illucens* meal per kilogram, diet TM – with 150 g of fish meal and 200 g of *Tenebrio molitor* meal per kilogram, diet ZM – with 150 g of fish meal and 200 g of *Zophobas morio* meal per kilogram.

Table S1. Analyzed fatty acid profile of experimental feeds for ide juveniles - continuation.

Fatty acids [g 100g fat^{-1}]	Diets			
	CON	HI	TM	ZM
C18:3 total amount	5.95	3.95	1.54	<0.5
C18:4n3 Stearidonic acid (SDA)	<0.5	<0.5	<0.5	<0.5
C20:2n6 Eicosadienoic acid	1.19	<0.5	<0.5	<0.5
C22:0 Behenic acid	<0.5	<0.5	<0.5	<0.5
C20:3n6 Dihomo-ɣ-linolenic acid	<0.5	<0.5	<0.5	<0.5
C22:1n11 Gadoleic acid	2.38	1.32	1.54	1.37
C22:1n9 Erucic acid	<0.5	<0.5	<0.5	<0.5
C22:1 total amount	2.38	2.63	1.54	1.37
C20:3n3 Eicosatrienoic acid	<0.5	<0.5	<0.5	<0.5
C20:4n6 Arachidonic acid	<0.5	<0.5	<0.5	<0.5
C23:0 Tricosylic acid	<0.5	<0.5	<0.5	<0.5
C22:2n6 Docosadienoic acid	<0.5	<0.5	<0.5	<0.5
C20:4n3 Eicosatetraenoic acid	<0.5	<0.5	<0.5	<0.5
C20:5n3 Eicosapentaenoic acid	2.38	1.32	1.54	<0.5
C24:0 Lignoceric acid	<0.5	<0.5	<0.5	<0.5
C24:1n9 Nervonic acid	<0.5	<0.5	<0.5	<0.5
C22:5n3 Docosadienoic acid	1.19	<0.5	<0.5	<0.5
C22:6n3 Docosahexaenoic acid	1.19	1.32	<0.5	<0.5
SFA	16.87	29.33	25.00	44.44
MUFA	54.22	48.00	56.25	41.67
PUFA	28.92	22.67	18.75	13.89
Omega-3	12.05	8.00	3.13	1.39
Omega-6	16.87	14.67	15.63	12.50
Omega-9	44.58	41.33	50.00	37.50

CON – diet with 300 g of fish meal per kilogram and no insect meals inclusion, HI – diet with 150 g of fish meal and 200 g of *Hermetia illucens* meal per kilogram, diet TM – with 150 g of fish meal and 200 g of *Tenebrio molitor* meal per kilogram, diet ZM – with 150 g of fish meal and 200 g of *Zophobas morio* meal per kilogram.

Table S2. Analyzed fatty acid profile of ide samples fed experimental diets.

Fatty acids [g 100g fat^{-1}]	Diets				Day zero
	CON	HI	TM	ZM	
C4:0 Butyric acid	<0.5	<0.5	<0.5	<0.5	<0.5
C6:0 Caproic acid	<0.5	<0.5	<0.5	<0.5	<0.5
C8:0 Caprylic acid	<0.5	<0.5	<0.5	<0.5	<0.5
C10:0 Capric acid	<0.5	<0.5	<0.5	<0.5	<0.5
C11:0 Undecylic acid	<0.5	<0.5	<0.5	<0.5	<0.5
C12:0 Lauric acid	<0.5	2.22	<0.5	<0.5	<0.5
C13:0 Tridecylic acid	0.64	0.74	0.76	0.78	1.85
C14:0 Myristic acid	1.91	2.22	2.29	1.55	1.85
C14:1 Myristoleic acid	<0.5	<0.5	<0.5	<0.5	<0.5
C15:0 Pentadecylic acid	<0.5	<0.5	<0.5	<0.5	<0.5
C15:1 Ginkgolic	<0.5	<0.5	<0.5	<0.5	<0.5
C16:0 Palmitic acid	19.75	18.52	19.08	20.16	14.81
C16:1n7 Palmitoleic acid	8.92	8.89	8.40	8.53	5.56
C16:1 total amount	8.92	8.89	8.40	8.53	5.56
C17:0 Margaric acid	<0.5	<0.5	<0.5	<0.5	<0.5
C16:2n4 Hexadecadienoic acid	<0.5	<0.5	<0.5	<0.5	<0.5
C17:1 Margaroleic acid	<0.5	<0.5	<0.5	<0.5	<0.5
C16:3n4 Hexadecatrienoic acid	<0.5	<0.5	<0.5	<0.5	<0.5
C18:0 Stearic acid	2.55	2.96	2.29	3.10	1.85
C18:1n9 Elaidic acid	<0.5	<0.5	<0.5	<0.5	<0.5
C18:1n9 Oleic acid	40.13	41.48	45.80	43.41	31.48
C18:1n7 Vaccenic acid	3.18	2.96	2.29	3.10	3.70
C18:1 total amount	43.95	45.93	48.85	47.29	35.19
C18:2n6 Linoleic acid	<0.5	<0.5	<0.5	<0.5	<0.5
C18:2 total trans amount	0.64	0.74	0.76	0.78	<0.5
C18:2 total amount	10.19	9.63	9.92	9.30	16.67
C18:2n6 Linoleic acid (LA)	9.55	8.89	9.16	8.53	16.67
C20:0 Arachidic acid	<0.5	<0.5	<0.5	<0.5	<0.5
C18:3n6 ɣ-Linolenic acid (GLA)	0.64	0.74	0.76	0.78	<0.5
C21:0 Heneicosylic acid	<0.5	<0.5	<0.5	<0.5	<0.5
C18:3n4 Octadecatrienoic acid	<0.5	<0.5	<0.5	<0.5	<0.5
C20:1n9 Eicosenoic acid	1.91	2.22	1.53	1.55	1.85
C20:1 total amount	2.55	2.22	1.53	2.33	3.70
C18:3n3 α-Linolenic acid (ALA)	2.55	1.48	0.76	0.78	3.70

CON – diet with 300 g of fish meal per kilogram and no insect meals inclusion, HI – diet with 150 g of fish meal and 200 g of *Hermetia illucens* meal per kilogram, diet TM – with 150 g of fish meal and 200 g of *Tenebrio molitor* meal per kilogram, diet ZM – with 150 g of fish meal and 200 g of *Zophobas morio* meal per kilogram, Day zero – a sample taken from all fish before allocating them to experimental groups.

Table S2. Analyzed fatty acid profile of ide samples fed experimental diets - continuation.

Fatty acids [g 100g fat^{-1}]	Diets				Day zero
	CON	HI	TM	ZM	
C18:3 total amount	2.55	2.22	1.53	1.55	3.70
C18:4n3 Stearidonic acid (SDA)	<0.5	<0.5	<0.5	<0.5	<0.5
C20:2n6 Eicosadienoic acid	0.64	0.74	<0.5	<0.5	1.85
C22:0 Behenic acid	<0.5	<0.5	<0.5	<0.5	<0.5
C20:3n6 Dihomo-ɣ-linolenic acid	0.64	0.74	0.76	0.78	<0.5
C22:1n11 Gadoleic acid	0.64	0.74	<0.5	0.78	1.85
C22:1n9 Erucic acid	<0.5	<0.5	<0.5	<0.5	<0.5
C22:1 total amount	0.64	0.74	<0.5	0.78	1.85
C20:3n3 Eicosatrienoic acid	<0.5	<0.5	<0.5	<0.5	<0.5
C20:4n6 Arachidonic acid	0.64	0.74	0.76	0.78	1.85
C23:0 Tricosylic acid	<0.5	<0.5	<0.5	<0.5	<0.5
C22:2n6 Docosadienoic acid	<0.5	<0.5	<0.5	<0.5	<0.5
C20:4n3 Eicosatetraenoic acid	<0.5	<0.5	<0.5	<0.5	<0.5
C20:5n3 Eicosapentaenoic acid	0.64	0.74	0.76	0.78	1.85
C24:0 Lignoceric acid	<0.5	<0.5	<0.5	<0.5	<0.5
C24:1n9 Nervonic acid	<0.5	<0.5	<0.5	<0.5	<0.5
C22:5n3 Docosadienoic acid	0.64	<0.5	<0.5	<0.5	<0.5
C22:6n3 Docosahexaenoic acid	1.91	1.48	0.76	0.78	3.70
SAFA	25.16	26.32	24.81	26.19	20.37
MUFA	56.77	57.89	60.47	58.73	46.30
PUFA	18.06	15.79	14.73	15.08	33.33
Omega-3	6.45	4.51	3.10	3.17	11.11
Omega-6	11.61	11.28	11.63	11.11	20.37
Omega-9	42.58	44.36	48.06	46.03	33.33

CON – diet with 300 g of fish meal per kilogram and no insect meals inclusion, HI – diet with 150 g of fish meal and 200 g of *Hermetia illucens* meal per kilogram, diet TM – with 150 g of fish meal and 200 g of *Tenebrio molitor* meal per kilogram, diet ZM – with 150 g of fish meal and 200 g of *Zophobas morio* meal per kilogram, Day zero – a sample taken from all fish before allocating them to experimental groups.
